# Treatment of loco-regional recurrence of nasopharyngeal carcinoma in a non-endemic area: oncologic outcomes, morbidity, and proposal of a prognostic nomogram

**DOI:** 10.3389/fonc.2023.1157584

**Published:** 2023-05-16

**Authors:** Vittorio Rampinelli, Marco Ferrari, Davide Mattavelli, Pierluigi Bonomo, Alessia Lambertoni, Mario Turri-Zanoni, Elisa D’Angelo, Daniela Alterio, Marco Cianchetti, Barbara Vischioni, Roberta Rosati, Michele Tomasoni, Marco Alparone, Stefano Taboni, Davide Tomasini, Marta Maddalo, Michela Buglione di Monale Bastia, Nicola Alessandro Iacovelli, Francesco Dionisi, Maurizio Bignami, Paolo Battaglia, Paolo Bossi, Alberto Deganello, Cesare Piazza, Alberto Schreiber, Piero Nicolai, Paolo Castelnuovo, Ester Orlandi

**Affiliations:** ^1^ Unit of Otorhinolaryngology – Head and Neck Surgery, ASST Spedali Civili, Department of Surgical and Medical Specialties, Radiological Sciences, and Public Health, University of Brescia, School of Medicine, Brescia, Italy; ^2^ Section of Otorhinolaryngology - Head and Neck Surgery, Department of Neurosciences, University of Padova, Padova, Italy; ^3^ Radiation Oncology, Azienda Ospedaliero-Universitaria Careggi, Florence, Italy; ^4^ Unit of Otorhinolaryngology and Head & Neck Surgery, Department of Biotechnology and Life Sciences, ASST Sette Laghi, University of Insubria, Varese, Italy; ^5^ Radiotherapy Unit, Azienda Ospedaliero Universitaria di Modena, Modena, Italy; ^6^ Division of Radiotherapy, IEO European Institute of Oncology, IRCCS, Milan, Italy; ^7^ Proton Therapy Unit, Azienda Provinciale Per i Servizi Sanitari, Trento, Italy; ^8^ Radiation Oncology Clinical Department, National Center for Oncological Hadrontherapy (CNAO), Pavia, Italy; ^9^ Department of Radiation Oncology, Brescia University, Brescia, Italy; ^10^ Radiotherapy 2 Unit, Fondazione IRCCS IstitutoTumori, Milan, Italy; ^11^ Department of Radiation Oncology, IRCCS Regina Elena National Cancer Institute, Rome, Italy; ^12^ Medical Oncology, Department of Medical and Surgical Specialities, Radiological Sciences, and Public Health, University of Brescia, Brescia, Italy; ^13^ Otolaryngology Head and Neck Surgery, IRCCS National Cancer Institute (INT), Milan, Italy

**Keywords:** nasopharyngeal carcinoma, salvage treatment, non-endemic cancer, recurrent tumor, proton therapy, IMRT

## Abstract

**Introduction:**

The study assessed outcomes and toxicities of different treatment modalities for local and/or regional recurrent nasopharyngeal carcinoma (NPC) in a non-endemic area.

**Methods:**

Patients treated with curative intent for recurrent NPC with salvage surgery, photon-based radiotherapy, proton therapy (PT), with or without chemotherapy, at different Italian referral centers between 1998 and 2020 were included. Adverse events and complications were classified according to the Common Terminology Criteria for Adverse Events. Characteristics of the patients, tumors, treatments, and complications are presented along with uni- and multivariate analysis of prognostic factors. A survival predictive nomogram is also provided.

**Results:**

A total of 140 patients treated from 1998 to 2020 were retrospectively assessed. Cases with lower age, comorbidity rate, stage, and shorter disease-free interval (DFI) preferentially underwent endoscopic surgery. More advanced cases underwent re-irradiation, fairly distributed between photon-based radiotherapy and PT. Age and DFI were independent factors influencing overall survival. No independent prognostic effect of treatment modality was observed. No significant difference in the morbidity profile of treatments was observed, with 40% of patients experiencing at least one adverse event classified as G3 or higher.

**Conclusion:**

Recurrent NPC in a non-endemic area has dissimilar aspects compared to its endemic counterpart, suggesting the need for further studies that can guide the choice of the best treatment modality.

## Introduction

Despite the intrinsic chemo-radiosensitivity and improvements in radiation techniques and systemic therapies, up to 20% of patients affected by nasopharyngeal carcinoma (NPC) experience persistent or recurrent loco-regional disease after primary radiotherapy (RT) with or without concomitant chemotherapy (1–3).

Unlike for primary treatment, there is no consensus on the therapeutic strategy to be adopted in the recurrent setting, with various options available. These are mainly represented by surgery, highly conformal RT techniques, such as intensity modulated RT (IMRT), stereotactic body RT (SBRT), and proton beam RT (PT), combined or not with concurrent chemotherapy (4, 5).

The recent literature and international recommendations seem to lean in favor of surgical treatment for resectable recurrences (4, 6, 7). Indeed, contemporary case series based on endoscopic surgery have reported similar-to-higher survival outcomes with lower morbidity compared with re-irradiation (re-RT) (8, 9). The only trial offering a head-to-head comparison of surgery vs. re-RT in the management of early-stage local recurrence of NPC demonstrated that endoscopic surgery significantly improved overall survival (OS) compared with IMRT (9). Furthermore, early timing of recurrence and sequelae of the primary treatment may hamper the possibility of curative re-RT.

However, heterogeneity of expertise in endoscopic surgery, variability in radiation oncologist experience and re-RT institutional volume, and lack of extensive knowledge in centers where the disease is not frequent, render the choice of salvage surgery vs. re-RT difficult, as the risk-benefit balance is rarely strongly in favor of one of the two options.

The concept of surgical resectability itself is subjective and frequently relative to the fact that a given procedure may be deemed as too invasive in view of patient’s general conditions and prognosis rather than referring to the genuine possibility to completely resect the tumor (10). Liu et al. considered lesions as resectable if limited to the nasopharyngeal cavity, nasal septum, superficial parapharyngeal space, or the base wall of the sphenoid sinus (9). However, these limits can be technically overcome. For instance, endovascular carotid closure or bypass enable the surgeon to extend the resection far laterally and posteriorly (10, 11). Moreover, despite advances in RT techniques, including IMRT, PT, and SRT, the survival benefit of re-RT is still offset by frequent fatal complications, making careful patient selection and re-RT planning and delivery even more mandatory (6). Finally, the availability and integration of other therapeutic options (i.e., neoadjuvant and adjuvant chemotherapy, PT, and immunotherapy) should also be considered in the decision-making process (12).

To further complicate the scenario, there are only few studies focusing on non-endemic NPC recurrence in the literature (13–16), and usually treatment protocols are based on the results of studies performed in endemic areas. Despite the similarities, specific non-endemic traits have been noticed, likely due to distinctive pathogenesis (14). Locoregional and distant recurrences seems to occur more and less frequently compared to endemic cohorts, respectively (13). Moreover, the survival and toxicity predictive models developed for endemic recurrences do not fit with the non-endemic counterpart, as highlighted by Boustani et al. (14). In light of the rarity of the clinical condition in non-typical areas, multicentric efforts should be done to clarify peculiarities and develop valid survival prognostic models.

The present paper analyzes the experience gathered by different referral centers in dealing with locally and/or regionally recurrent/persistent NPC in a non-endemic area, with the aim of analyzing treatment modalities, oncologic and morbidity outcomes, and prognostic factors. Survival predictive nomograms are herein also provided.

## Materials and methods

In this multi-institutional, retrospective study, we included locally and/or regionally recurrent/persistent NPC patients, treated with curative intent with salvage surgery or definitive RT at eight Italian referral centers.

The study period was between November 1998 and January 2020. Inclusion criteria were the following: a) having received a curative photon-based treatment for the primary NPC, at a prescribed total dose of at least of 63 Gy with conventional fractionation (corresponding to a biological effective dose (BED) of at least 74.34 Gy (α/β = 10, BED10) and an Equivalent Dose in 2Gy fractions (EQD2) of 61.95 Gy); b) histologically or cytologically-proven local, regional or loco-regional recurrent/persistent NPC confirmed after physical examination and radiological imaging [computed tomography (CT) or magnetic resonance imaging (MRI), and/or positron emission tomography with 2-deoxy-2-[fluorine-18]fluoro-D-glucose/CT (^18^F-FDG PET/CT)]; c) first and in field recurrences following primary treatment;d) follow-up after salvage therapy of at least 3 months.

Persistent disease was defined as residual disease detected at the first re-staging imaging after completion of (chemo-)RT, within a period of 6 months. Conversely, recurrent disease was defined as relapse beyond 6 months after definitive therapy with radiologic evidence of complete clinical response to primary treatment (3). Recurrent NPCs were classified following the 8^th^ Edition of the TNM staging system (17).

The choice of the treatment between (neo)adjuvant chemotherapy, salvage surgery, and/or re-RT, was based on tumor and patient characteristics as well as expertise of the local multidisciplinary team. Surgery was exclusively performed *via* nasopharyngeal endoscopic resection (NER) and/or neck dissection. Curative re-RT could be delivered in postoperative or definitive setting in the form of IMRT (including volumetric modulated arc therapy, VMAT), PT or SBRT. SBRT included CyberKnife or VMAT with high-precision imaged-guided system. Brachytherapy (BRT) with High Dose Rate (HDR) or Pulsed Dose Rate (PDR) with ^192^Ir alone or in combination with photon-based RT was included as treatment strategy. The minimum overall total dose had to be at least 31 Gy and 31 Gy Relative Biological Effectiveness (RBE) EQD2 with photon-based and proton-based approach, using conventional or hypofractionated regimens, respectively.

The following anonymized data were extracted from institutional databases:

- Patient-related variables: age, gender, comorbidities (number, Charlson comorbidity index [CCI]);
- Tumor-related variables (type of relapse, disease-free interval [DFI], histology);
- Treatment-related variables ([neo-]adjuvant treatments, characteristics of surgery and/or re-RT with or without concomitant chemotherapy);
- Adverse events and complications related to salvage treatments were classified according to the Common Terminology Criteria for Adverse Events [CTCAE v5.0] (18).

### Statistical analysis

Statistical analysis was performed using RStudio (2022.07.2). Variables assessed in the study were reported with standard descriptive statistics: continuous variables were summarized as median, range, and interquartile range, whereas categorical variables as absolute and percentage distributions. Contingency tables were used to assess the relationship between primary T category and T category at recurrence, and the relationship between clinical and pathological TN categories in patients who underwent salvage surgery. Fisher’s exact test, chi-square test or Mann-Whitney test, as appropriate, was used to compare rates of complications relative to the treatment strategy (classified as surgery, re-RT, and combination thereof) and cumulative RT dose.

Survival analysis was conducted considering OS as the primary outcome, and disease-specific (DSS), recurrence-free (RFS), local recurrence-free (LRFS), regional recurrence-free (RRFS), and distant recurrence-free survival (DRFS) as secondary outcomes. Time-to-event observations were determined based on time from diagnosis of recurrence to event occurrence or censor. Events were defined as follows: death of any cause for OS, disease-specific death for DSS, further recurrence for RFS, and further local, regional, or distant recurrence for LRFS, RRFS, and DRFS, respectively. Univariate prognostic analysis was performed with the log-rank test for categorical variables and univariate Cox proportional hazards model for continuous variables. Multivariable analysis was performed with a Cox proportional hazards model. Selection of variables to be included in the model was made *a priori* based on clinical relevance of each factor according to the authors’ personal experience. Moreover, variables not selected *a priori* and exhibiting a prognostic effect at univariate analysis were also considered to build the multivariable model. Assumptions of the Cox proportional hazards model were checked as follows: proportional hazards assumption was tested through the global Schoenfeld test, influential observations were checked through deviance residual analysis, and non-linearity was assessed (when needed) by Martingale residual analysis. Multi-collinearity of covariates was assessed with a multi-collinearity test; covariates with a variance inflation factor of 5 or higher were considered as multi-collinear and were excluded from the model. A nomogram predicting OS at 1, 2, 5, and 10 years was created and internally validated at each time point through a 300-repetition bootstrap. Calibration graphs were obtained using the Akaike’s Information Criterion as stopping rule. Internal validation was completed by calculating the C-index.

A 4-state multistate model was created, including the following states: alive with neither ≥G3 toxicity nor recurrence, alive with ≥G3 toxicity, alive with recurrence (regardless of the presence of ≥G3 toxicity), dead (absorbing state). A multivariable analysis was performed to identify factors independently favoring transitions from one to another state.

Level of significance was set a 0.05; p values between 0.05 and 0.10 were highlighted with the term “close-to-significant” as not formally significant, but potentially marking clinically relevant associations.

## Results

The study included 140 patients. Patients and tumor characteristics are summarized in **Table 1**. Considering the treatment of the primitive cancer, 99/140 (70.7%) patients underwent concomitant chemo-radiotherapy, the remaining 41/140 (29.3%) only radiotherapy.

**Table 1:** Patient and tumor characteristics.

**Gender**	Male: 102/140 (72.9%) Female: 38/140 (27.1%)
**Ethnicity**	Caucasian: 134/140 (95.7%) Asian: 3/140 (2.1%) African: 3/140 (2.1%)
**Dose of the first RT course (Average; Median; Range)**	69.0; 70.0; 63-72 Gy
**Age at recurrence date (Average; Median; Range)**	52.3; 51.0; 25-81
**Comorbidities at time of recurrence**	51.5%
**Charlson Comorbidity Index (Average; Median; Range)**	4.2; 3; 2-16
**Type of relapse**	Recurrence: 128/140 (91.5%); Persistence: 12/140 (8.5%) Local: 108/140 (77.1%) Regional: 7/140 (5.0%) Locoregional: 24/140 (17.1%) NA: 1/140 (0.1%)
**DFI (Average; Median; Range)**	45.4; 23; 3-316
**rcT (TNM VIII edition)**	T0: 7/140 (5.0%) T1: 40/140 (28.6%) T2: 24/140 (17.1%) T3: 31/140 (22.1%) T4: 34/140 (24.3%) NA: 4/140 (2.8%)
**rcN (TNM VIII edition)**	N0: 108/140 (77.1%) N1: 13/140 (9.3%) N2: 10/140 (7.1%) N3: 3/140 (2.1%) NA: 6/140 (4.3%)
**rcStage**	I: 38/140 (27.1%) II: 21/140 (15.0%) II: 36/140 (25.7%) IVA: 36/140 (25.7%) IVB: 0/140 (0.0%) NA: 9/140 (6.4%)
**Nodal levels involved at recurrence**	I: 1/22 (4.5%) II :15/22 (68.2%) III: 5/22 (22.7%) IV: 2/22 (9.0%) V: 1/22 (4.5%) Retropharyngeal: 7/22 (31.8%) NA: 9/31 (29.0%)
**Histology of recurrence**	Keratinizing NPC: 14/123 (11.4%) Non-keratinizing differentiated NPC: 2/123 (1.6%) Non-keratinizing undifferentiated NPC: 106/123 (86.2%) Basaloid NPC: 1/123 (0.8%) NA: 17/140 (12.1%)

DFI, Disease free interval; NA, Data not available; NPC, Nasopharyngeal carcinoma.

EBER (Epstein-Barr virus (EBV)-encoded small RNA) status was not systematically assessed (data available for 65 of 140 patients). When tested, it was found positive at pretreatment biopsy or definitive histological examination in all but one patient (98.5%). A substantial proportion of recurrent tumors (53.7%) showed a different rT category compared with the cT category at presentation. The relationship of T category at primary presentation vs. T category at recurrence is detailed in **Table 2**. In the subset of patients who underwent surgery as part of their salvage treatment, a considerable match between the clinical and pathological rT category was observed (95.0%). On the contrary, a tendency towards over-diagnosis of nodal involvement was demonstrated (80.5% of tumors classified as rcN+ resulted rpN0). The relationship of rcTN category relative to rpTN category in surgically treated patients is detailed in **Table 3**.

**Table 2:** Contingency table showing the relationship of T category at primary presentation versus T category at recurrence.

Data available for 123 patients		rcT classification				
		T0	T1	T2	T3	T4
**cT classification**	**T1**	2	22	7	6	7
	**T2**	2	6	9	3	5
	**T3**	0	3	3	11	5
	**T4**	1	6	3	7	15

**Table 3:** Contingency table showing the relationship of rcTN category relative to rpTN category in surgically treated patients.

Data available for 60 patients		rpT classification				
		T0	T1	T2	T3	T4
**rcT** **classification**	**T0**	3	0	0	0	0
	**T1**	0	23	2	1	0
	**T2**	0	0	8	0	0
	**T3**	0	0	0	20	0
	**T4**	0	0	0	0	3
Data available for 71 patients		**rpN classification**				
		**N0**	**N1**	**N2**	**N3**	
**rcN classification**	**N0**	28	1	1	0	
	**N1**	18	1	0	0	
	**N2**	13	5	1	0	
	**N3**	2	1	0	0	

### Treatment

**Figure 1:**
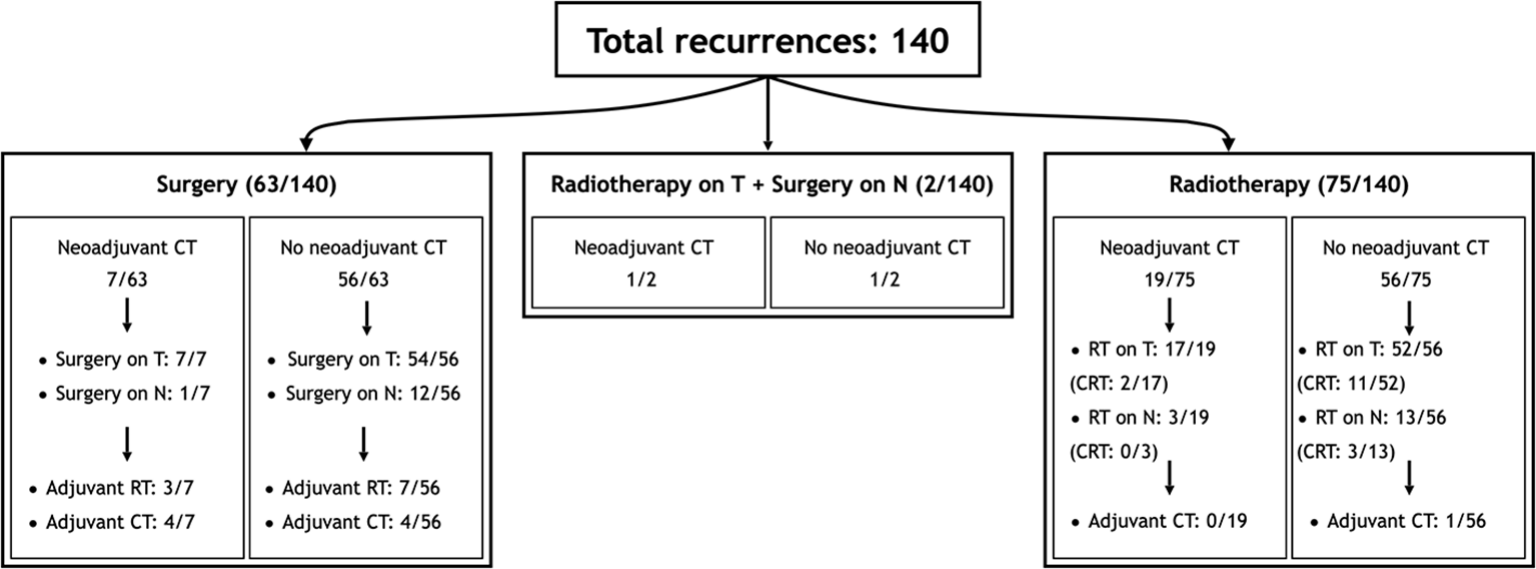
Scheme of patient distribution in treatment subgroups. CT, Chemotherapy; CRT, Radiotherapy with concomitant chemotherapy; RT, Radiotherapy.

Sixty-five (46.4%) patients received surgery as part of their salvage treatment, whereas the remaining 75 (53.6%) underwent a non-surgical treatment. Neoadjuvant chemotherapy was included in the treatment in 27 (19.3%) patients, whereas adjuvant chemotherapy in 9 (6.4%). **Figure 1** and **Tables 4**, **5** summarize the scheme of treatment and details of each treatment approach. Median prescription RT dose of the first RT course was 70 Gy (84 Gy BED10 and 70 GyEQD2) both for recurrent patients subsequently treated with postoperative re-irradiation and definitive re-irradiation. Adjuvant re-RT was mainly given in case of close or positive margins after surgery (8/10 patients, 80%), with median prescribed radiation dose of 54 Gy (64.8Gy BED10 and 54 GyEQD2) and 58 GyRBE (69.6 GyRBE BED10 and 58 GyRBE EQD2) delivered through IMRT/VMAT and PT, respectively. No patients received re-irradiation after neck dissection. Definitive re-RT was mostly delivered on primary tumor site, less commonly on unresectable retropharyngeal nodes, with median prescribed radiation dose of 56 Gy (67.2 Gy BED10 and 56 GyEQD2) and 54 GyRBE (64.8GyRBE BED10 and 54 GyRBE EQD2) with IMRT/VMAT and PT, respectively. In this setting, 7/77 patients (9.1%) received SBRT with a median prescribed radiation dose of 25 Gy (37.5 Gy BED10 and 31.25 GyEQD2). Only 1 of these 7 patients received a lower SBRT dose (20 Gy, 30 Gy BED10, 25 GyEQD2). Two of 77 (2.6%) patients received PDR-BRT as a boost after a first phase of IMRT. Data about SBRT doses were not available.

**Table 4:** Details of patient distribution in treatment subgroups.

Patients treated with salvage surgery ± RT (63/140)*	Patients treated with definitive RT (75/140)
Neoadjuvant CT: 7/63 Surgery on T: 61/63 Surgery on N: 13/63 Adjuvant CT: 8/63 Adjuvant RT: 10/63	Neoadjuvant CT: 19/75 Concomitant CT: 16/75 RT on T: 69/75 RT on N: 16/75 Adjuvant CT: 1/75
DFI (median): 18 months Age (median): 51 years Charlson Comorbidity Index (median): 1 rcT distribution: 44.1% T1, 15.5% T2, 35.6% T3, and 5.0% T4	DFI (median): 23 months Age (median): 54 years Charlson Comorbidity Index (median): 2 rcT distribution: 20.6% T1, 22.1% T2, 14.7% T3, and 42.6% T4.

*2/140 Patients underwent RT on T and surgery on N, with neoadjuvant CT in 1 case.

DFI, Disease free interval; CT, Chemotherapy; RT, Radiotherapy.

**Table 5:** Treatment details.

**Surgery**	**Type of surgery on T**	NER type 1: 6/61 (9.8%) NER type 2: 14/61 (22.9%) NER type 3: 41/61 (67.2%)
	**Reconstruction**	None: 36/61 (59.0%) TPFF: 6/61 (9.8%) NSF: 19/61 (31.1%)
	**Margin status**	R0: 52/61 (85.2%) R1: 8/61 (13.1%) R2: 1/61 (1.6%)
**Surgery + re-irradiation**	**Radiotherapy technique**	IMRT/VMAT: 8/10 (80.0%) PT: 2/10 (20.0%)
	**Prescribed radiation doses (Median; Range)**	IMRT/VMAT: 54; 54-56 Gy PT: 58; 46-66 GyRBE
	**Radiation fractionation (Median; Range)**	IMRT/VMAT: 2; 2-2 Gy PT: 2; 1.8-2 GyRBE
**Definitive re-irradiation**	**Radiotherapy technique**	IMRT/VMAT: 35/77(45.5%) SBRT: 7/77 (9.1%) PT: 33/77 (42.8%) Brachytherapy: 2/77 (2.6%)
	**Prescribed radiation doses (Median; Range)**	IMRT/VMAT: 56:54-72 SBRT: 25; 20-35 Gy PT: 54; 45-70 GyRBE Brachytherapy (as boost): NA
	**Radiation fractionation (Median; Range)**	IMRT/VMAT: 2; 1.2-2 Gy SBRT: 5; 3-5 Gy PT: 2; 1.8-3 GyRBE
**Neoadjuvant chemotherapy**	**Response to neoadjuvant chemotherapy**	CR: 1/27 (5.0%) PR: 7/27 (35.0%) SD: 8/27 (40.0%) PD: 4/27 (20.0%) NA: 7/27 (31.3%)

CR, Complete response; IMRT, Intensity modulated radiotherapy; NA, Data not available; NER, Nasopharyngeal endoscopic resection; NSP, Nasoseptal flap; PD, Progression of disease; PR, Partial response; PT, Proton therapy; RT, Radiotherapy; SBRT, Stereotactic body radiotherapy; SD, Stable disease; TPFF, Temporoparietal fascial flap; VMAT, Volumetric modulated arc therapy.

Taking into account both the first RT course and photon- and proton-based re-irradiation (excluding BRT), median cumulative prescribed total dose was 126 GyRBE (151.2GyRBE BED10, 126 GyRBE EQD2) and 115 GyRBE (140GyRBE BED10, 115 GyRBE EQD2) in postoperative and definitive settings, respectively.

### Survival outcomes

Median duration of follow-up was 29 months [range, 3-160; interquartile range (IQR), 17-63]. At last examination, patients’ status was as follows: 67 (47.8%) were alive with no evidence of disease, 22 (15.7%) alive with disease, 46 (32.8%) dead of disease, and 5 (3.6%) dead of other cause. Further recurrences following the first salvage treatment occurred preferentially at the local site. Of a total of 59 further relapses, 38 recurred again at the local sites, 16 on regional lymph nodes, and 5 on both.

**Figure 2:**
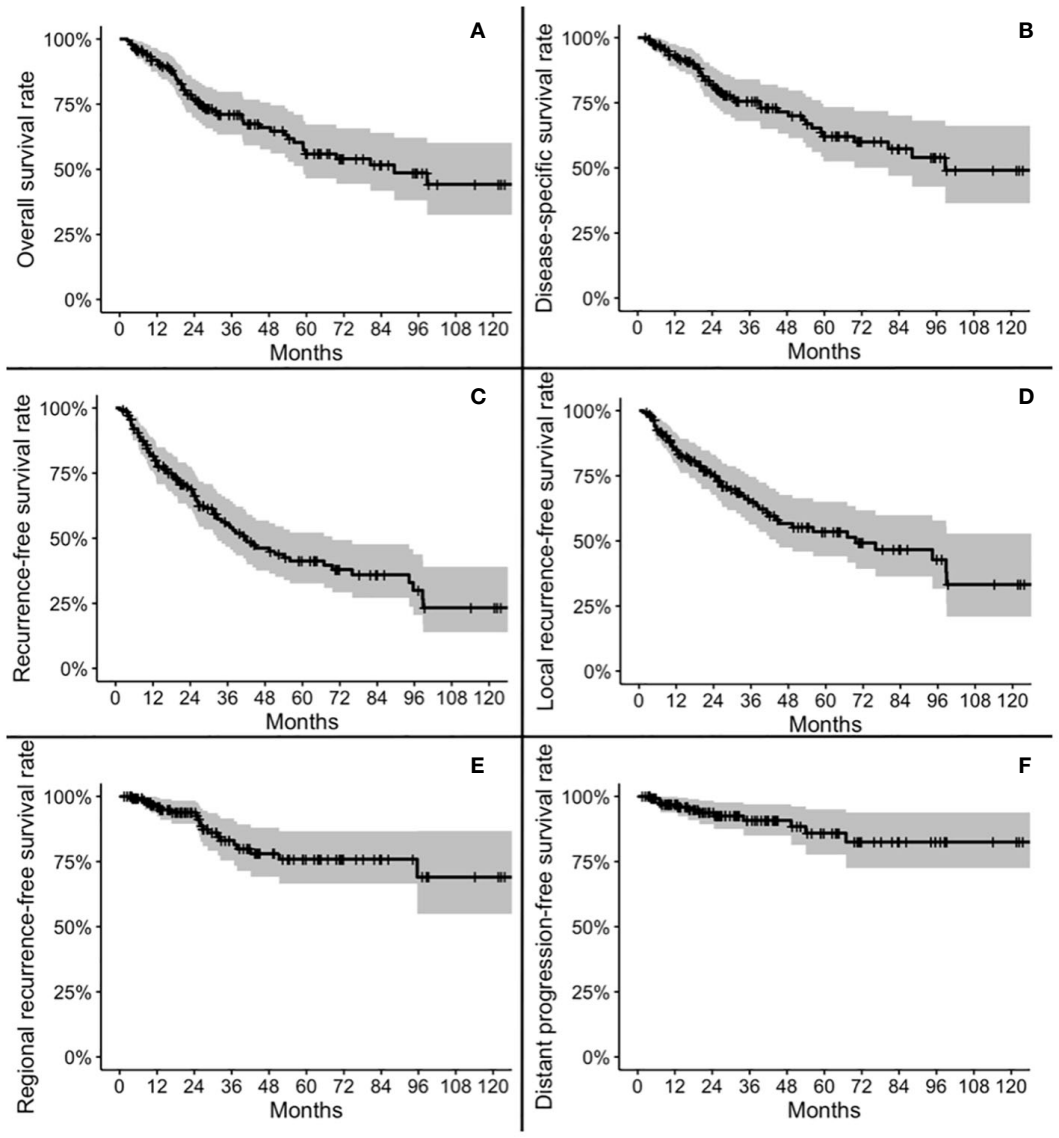
Kaplan-Meier plots for overall survival **(A)**, disease-specific survival **(B)**, recurrence-free survival **(C)**, local recurrence-free survival **(D)**, regional recurrence-free survival **(E)**, and distant progression-free survival **(F)**.

Five-year OS, DSS, RFS, LRFS, RRFS, and DRFS were 55.9, 62.1, 41.3, 53.5, 75.9, and 85.9%, respectively. Ten-year OS, DSS, RFS, LRFS, RRFS, and DRFS were 44.2, 49.1, 23.3, 33.3, 69.0, and 82.5%, respectively (**Figure 2**).

### Univariable survival analysis

At univariable analysis, cT, rcT, and stage at recurrence were factors influencing OS and DSS. Histology, cT, cN, rcT, stage at recurrence, and type of salvage treatment were factors influencing RFS. Margin status influenced both DSS and RFS (**Table 6**).

**Table 6:** Univariate prognostic analysis of patient-, cancer-, and treatment-related variables on overall survival (OS), disease-specific survival (DSS), and recurrence-free survival (RFS).

Variable	5-year OS	P-value	5-year DSS	P-value	5-year RFS	P-value
Gender	Male: 50.7% Female: 58.7%	0.10	Male: 57.7% Female: 62.6%	0.21	Male: 36.3% Female: 58.8%	0.21
Histology	Keratinizing: 53.2% Non−keratinizing differentiated: 60.0%* Non−keratinizing undifferentiated: 55.7%	0.39	Keratinizing: 65.1% Non−keratinizing differentiated: 60.0%* Non−keratinizing undifferentiated: 61.7%	0.57	Keratinizing: 34.0% Non−keratinizing differentiated: 22.9%* Non−keratinizing undifferentiated: 44.9%	**0.014**
cT of primary tumor	T1: 63.1% T2: 84.4% T3: 35.8% T4: 48.3%	**0.047**	T1: 72.2% T2: 87.9% T3: 41.1% T4: 52.0%	**0.043**	T1: 49.0% T2: 59.2% T3: 26.4% T4: 28.6%	**0.038**
cN of primary tumor	N0: 67.9% N1: 16.5% N2: 52.5% N3: 50.0%*	0.49	N0: 79.1% N1: 20.7% N2: 60.5% N3: 50.0%*	0.29	N0: 62.2% N1: 41.3%* N2: 30.8% N3: 33.3%*	**0.0031**
Stage of primary tumor	I: 66.7% II: 79.3% III: 47.5% IV: 33.2%	0.06	I: 100% II: 79.3% III: 53.3% IV: 37.3%	0.066	I: 76.2% II: 50.0% III: 31.4% IV: 19.2%	0.051
rcT	T0: 64.3% T1: 87.6% T2: 61.0% T3: 39.3% T4: 39.4%	**0.0021**	T0: 85.7% T1: 93.1% T2: 64.2% T3: 46.7% T4: 41.1%	**0.00053**	T0: 28.6% T1: 72.0% T2: 31.7% T3: 35.2% T4: 26.6%	**0.00013**
rcN	N0: 57.7% N1: 49.1% N2: 50.0%* N3: 66.7%	0.68	N0: 63.8% N1: 61.4% N2: 50.0%* N3: 66.7%	0.35	N0: 46.4% N1: 23.9%* N2: 25.0%* N3: 33.3%	0.5
rStage	I: 81.7% II: 53.6% III: 37.6% IV: 45.3%	**0.0048**	I: 92.9% II: 59.6% III: 44.3% IV: 47.2%	**0.00079**	I: 71.3% II: 35.6%* III: 30.5% IV: 31.7%	**<0.0001**
Primary tumor treatment	RT: 57.0% CRT: 55.9%	0.47	RT: 68.0% CRT: 60.7%	0.56	RT: 44.7% CRT: 30.1%	0.23
Recurrent tumor treatment	Surgery: 65.6% (C)RT: 48.7% Surgery + (C)RT: 51.1%	0.22	Surgery: 72.5% (C)RT: 55.5% Surgery + (C)RT: 51.1%	0.13	Surgery: 59.1% (C)RT: 28.9% Surgery + (C)RT: 40.9%	**0.0037**
Chemotherapy for recurrent tumor (induction and/or adjuvant)	Yes: 56.7% No: 55.4%	0.73	Yes: 60.2% No: 63.1%	0.94	Yes: 39.8% No: 45.5%	0.82
Margin status	R0: 64.5% R1: 46.7%	0.16	R0: 71.3% R1: 46.7%	**0.048**	R0: 60.4% R1: 40.0%	**0.0035**

*3-year estimate; RT, Radiotherapy; CRT, Radiotherapy with concomitant chemotherapy; CRT - Radiotherapy with or without concomitant chemotherapy.Words in bolds are statistically significant.

### Multivariable and multistate survival analysis

At multivariable analysis, age and DFI were independent factors negatively influencing OS and DSS (i.e., higher age/DFI were associated with worse prognosis), in contrast to stage and treatment modality. Primary regional disease, regional recurrence, and induction/adjuvant chemotherapy were independent factors influencing RFS (**Table 7**). In patients who received local surgery, margin status was not an independent factor affecting RFS.

**Table 7:** Multivariable prognostic analysis of patient-, cancer-, and treatment-related variables on overall survival (OS), disease-specific survival (DSS), and recurrence-free survival (RFS).

Variable	OS		DSS		RFS	
	HR (95% CI)	p	HR (95% CI)	p	HR (95% CI)	p
**Age**	1.06 (1.02, 1.09)	**<0.001**	1.04 (1.00, 1.08)	**0.03**	/	/
**DFI**	1.01 (1.00, 1.01)	**0.02**	1.01 (1.00, 1.02)	**0.04**	1.00 (0.99, 1.00)	0.35
cT8						
cT1 or cT2	Reference		Reference		Reference	
cT3 or cT4	1.76 (0.88, 3.53)	0.11	2.04 (0.93, 4.47)	0.08	2.04 (1.12, 3.69)	**0.019**
cN8						
cN0	Reference		Reference		Reference	
cN+	1.56 (0.81, 3.00)	0.18	1.92 (0.91, 4.03)	0.09	2.43 (1.36, 4.34)	**0.003**
rcT8						
rcT0	Reference		Reference		Reference	
rcT1	0.65 (0.10, 4.30)	0.66	0.52 (0.05, 5.36)	0.58	0.22 (0.04, 1.08)	0.06
rcT2	2.29 (0.46, 11.49)	0.31	2.92 (0.44, 19.49)	0.27	0.79 (0.21, 2.98)	0.72
rcT3	2.42 (0.48, 12.20)	0.28	2.73 (0.42, 17.55)	0.29	0.81 (0.20, 3.20)	0.76
rcT4	3.71 (0.80, 17.22)	0.09	5.82 (1.00, 33.90)	**0.05**	0.78 (0.22, 2.78)	0.70
rcN8						
rcN0	Reference		Reference		Reference	
rcN+	1.47 (0.65, 3.33)	0.36	1.74 (0.74, 4.07)	0.20	2.08 (1.00, 4.34)	**0.05**
TREATMENT OF RECURRENCE						
Endoscopic surgery*	Reference		Reference		Reference	
Re−irradiation	0.94 (0.38, 2.34)	0.89	1.02 (0.35, 3.00)	0.97	1.89 (0.79, 4.53)	0.15
Endoscopic surgery + re−irradiation*	1.01 (0.33, 3.10)	0.99	1.71 (0.51, 5.81)	0.39	1.10 (0.37, 3.26)	0.85
CHEMOTHERAPY						
Neither nCT nor aCT	Reference		Reference		Reference	
nCT and/or aCT	0.70 (0.36, 1.38)	0.30	0.66 (0.32, 1.38)	0.27	0.52 (0.29, 0.94)	**0.031**

aCT, adjuvant chemotherapy; DFI, Disease free interval; nCT, neo-adjuvant chemotherapy. *In the multivariable model, no significant difference of the outcomes was observed according to margin status in patients receiving surgery.Words in bolds are statistically significant.

**Figure 3:**
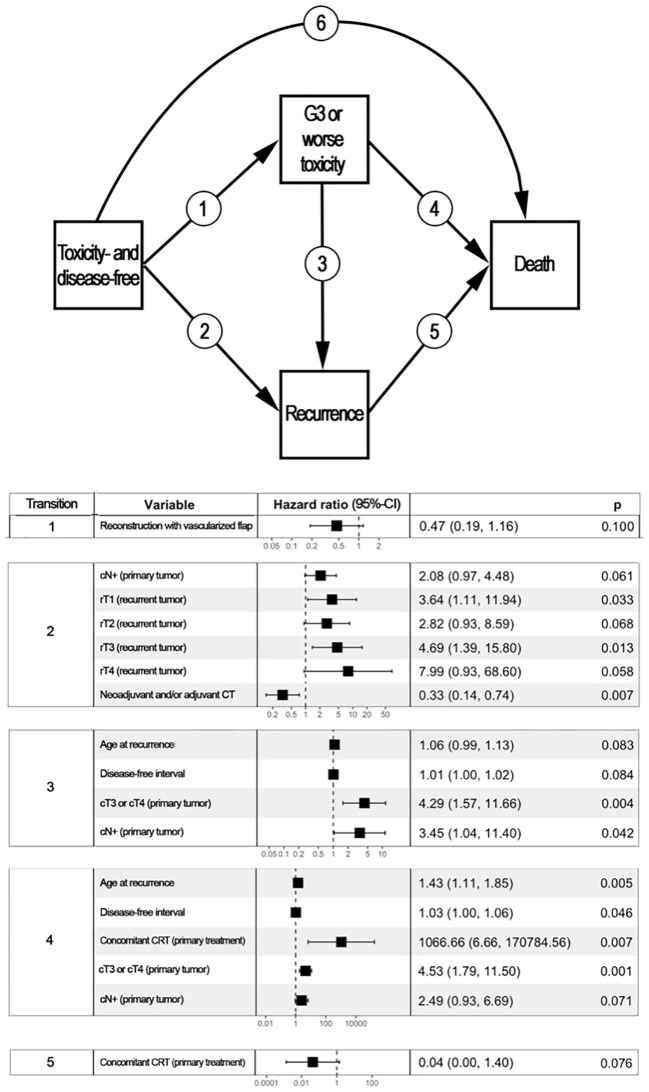
Multistate multivariable analysis. The upper part of the figure represents graphically the 4-state, 6-transition model adopted to analyze the post-treatment course of patients included in the study. Each box represents a state, whereas arrows represent the potential transitions conceived in the model. The lower part of the figure displays a table reporting variables with a statistically significant or close-to-significant impact on state transition. For instance, disease- and toxicity-free patients who underwent a reconstruction based on vascularized tissue as part of their re-treatment had a close-to-significantly lower chance of developing ≥G3 toxicity (transition 1, p=0.100). patients with a nasopharyngeal carcinoma classified as cT3/4 at primary presentation who had a ≥G3 toxicity were more likely to either develop a recurrence (transition 3, p=0.004) or die being disease-free (transition 5, p=0.001) when compared to subjects with cT1/2 primary nasopharyngeal cancer. Transition 6 was not associated with any factor included in the analysis. CI, Confidence interval; CRT, Radiotherapy with concomitant chemotherapy; CT, Chemotherapy.

**Figure 4:**
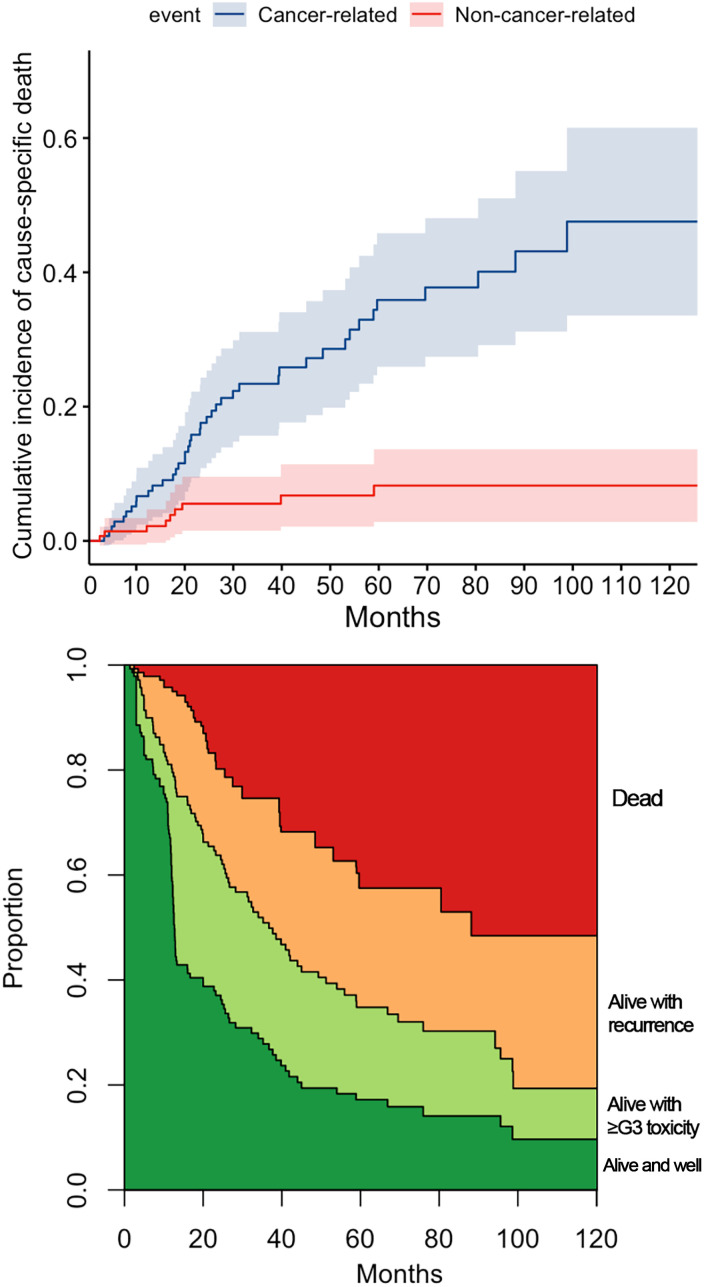
The cumulative incidence plot for cancer and non-cancer-related death. The stacked plot depicts the proportion of state (dead, alive with recurrence, alive with ≥G3 toxicity, or alive and well) distribution in the timepoint.

**Figure 3** reports the variables with a statistically significant or close-to-significant impact on status transition at multistate multivariable analysis. In particular, the reconstruction with a vascularized flap displayed a protective effect on the development of ≥G3 toxicity (transition 1 in **Figure 3**). N category at presentation and rT category significantly affected the risk of developing a recurrence from a toxicity- and disease-free state (transition 2 in **Figure 3**). On the contrary, use of neoadjuvant and/or adjuvant chemotherapy in the salvage treatment was associated with a protective role towards transition 2. Age, DFI, and TN categories at primary presentation affected the transition between a ≥G3 toxicity state to recurrence (transition 3 in **Figure 3**). Age, DFI, primary treatment including concomitant chemotherapy, and TN categories of the primary lesion were associated with increased risk of death from a ≥G3 toxicity state (transition 4 in **Figure 3**). Primary treatment including concomitant chemotherapy was a protective factor with regards to transition from recurrence to death (transition 5 in **Figure 3**). The cumulative incidence standard and stacked plot (**Figure 4**) suggest that cancer-specific mortality increased constantly in the first 5 years after salvage treatment, while non-cancer related deaths are concentrated in the first 2 years. Treatment-related adverse events (status alive with ≥G3 toxicity) occurred mainly in the first year.

**Figure 5:**
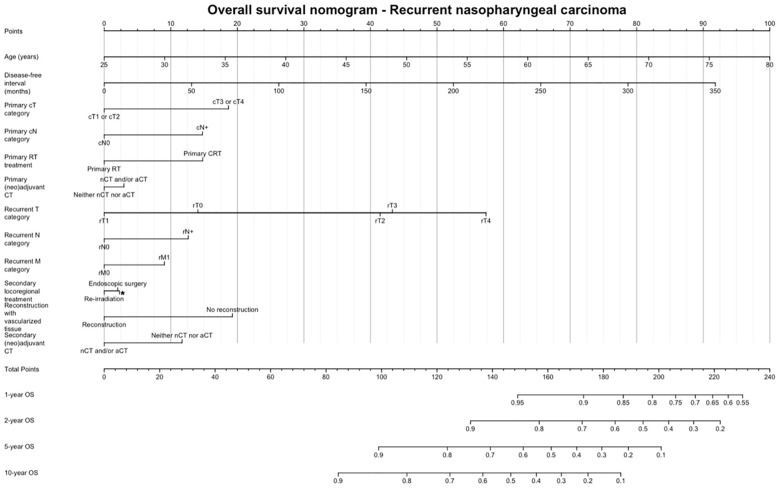
Overall survival nomogram, estimating the probability of survival at 1, 2, 5, and 10 years after retreatment. CT, Chemotherapy; RT, Radiotherapy.

**Figure 6:**
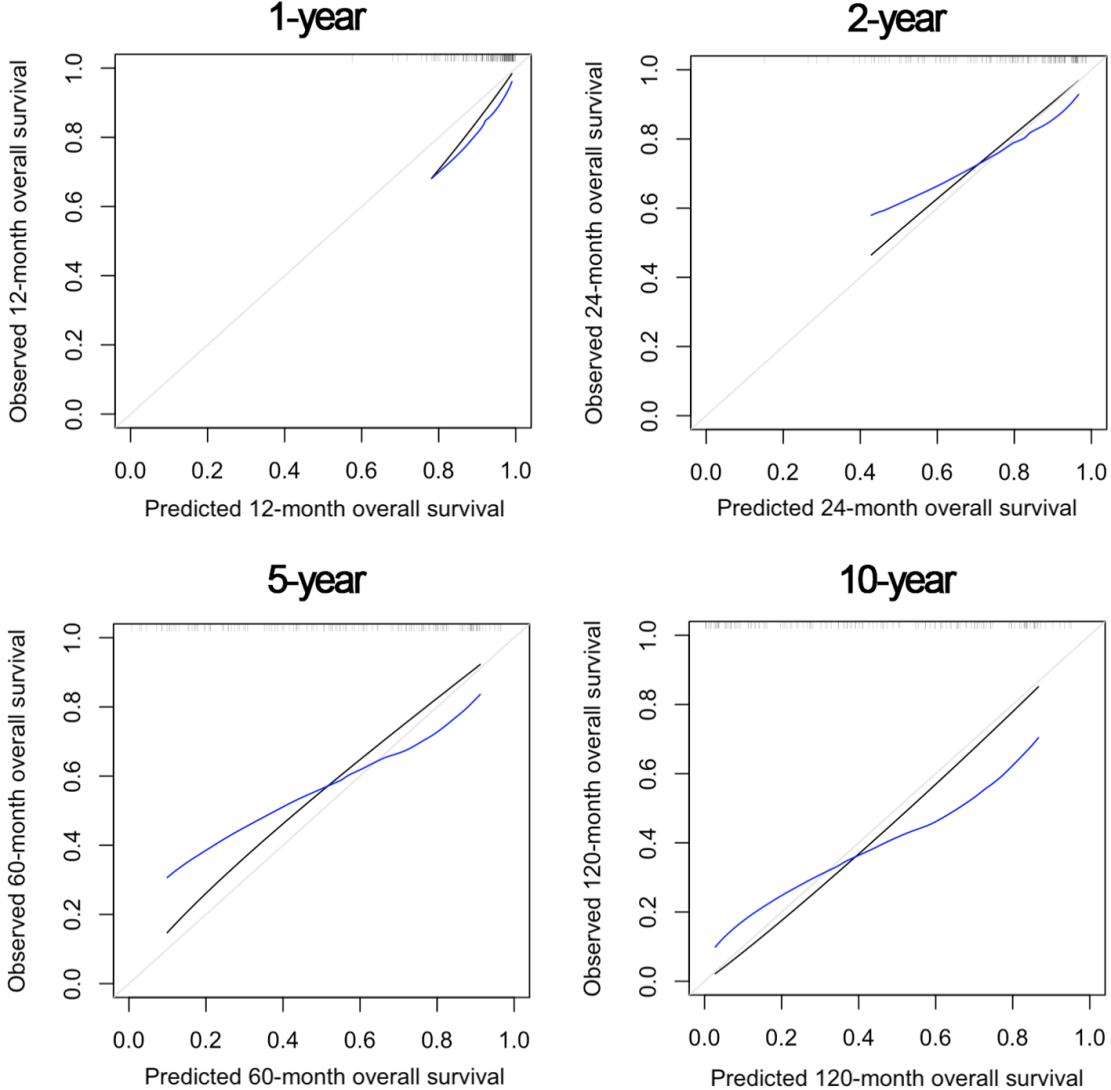
Internal validation of the nomogram model at 1, 2, 5, and 10 years after re-treatment timepoints, showing satisfactory predictive performance.

**Figure 5** shows the OS nomogram, which allows estimation of the probability of survival at 1, 2, 5, and 10 years after retreatment. Internal validation of the model showed satisfactory performance of the nomogram (C-index: 0.732) (**Figure 6**).

### Treatment toxicity

Fifty-six (40%) patients experienced at least one adverse event classified as ≥G3; the rate of ≥G3 toxicity was 40.7, 54.5, and 37.3% in the cohorts treated with surgery, surgery + re-RT, and re-RT, respectively (p=0.520). **Table 8** shows the spectrum of complications classified as ≥G3 observed in the series. The rate of ≥G3 toxicity events was equally frequent in patients who received neoadjuvant chemotherapy (28.6%) and those who did not (29.8%, p=1.000). The total cumulative dose was not statistically different in patients who developed a ≥G3 toxicity event (median: 117 GyRBE) compared with those who did not (median: 110 GyRBE, p=0.662).

**Table 8:** Spectrum of complications classified as ≥G3 observed in the series.

	Surgery	Surgery + postoperative Re-irradiation	Definitive Re-irradiation
Intraoperative			
Internal carotid artery injury	1	0	0
Early post-treatment (≤30 days)			
Osteomyelitis	0	0	1
Pain	3	1	1
Mucositis	0	0	6
XII nerve palsy	1	0	1
Extraocular muscle paresis	1	1	0
Dysphagia	0	0	1
Neutropenia	0	0	1
Late post-treatment (>30 days)			
Osteomyelitis/osteoradionecrosis	6	2	10
Nasal congestion	3	1	0
Pain	5	3	0
Mucositis	0	2	2
Internal carotid artery blowout	0	0	3
Brain edema	0	1	2
Brain necrosis	1	0	2
Trismus	3	0	1
Hearing loss	1	0	5
Dyspnea	3	0	0

## Discussion

The present paper collects the experience of different referral centers in dealing with local and/or regional persistence or recurrence of NPC in a non-endemic area, with the aim of analyzing treatment modalities, prognostic factors, and morbidity outcomes. Almost all large NPC series, in fact, are from endemic areas in Asia, with the literature from non-endemic populations being based on heterogeneous and small cohorts (13). Furthermore, these frequently present a share of immigrants from endemic areas that inflates the results of the analysis (13). In our cohort, more than 95% of patients belonged to Caucasian ethnicity, which makes it, to the best of our knowledge, the most homogeneous clinical series of non-endemic recurrent NPC in the current literature.

We observed an average age at recurrence of 52.3 years and a median DFI of 23 months, widely ranging between 3 and 316 months. This observation is in line with the literature coming from endemic areas (3, 14, 19). Of note, more than half of the recurrences in the present study (i.e., candidate to salvage treatment) occurred in the first 2 years after primary (chemo-)RT, which suggests that follow-up strategies should be particularly focused during that time span. On the other hand, the remaining half of recurrences were observed over a wide time frame after 2 years, with the latest representing most likely secondary cancers. This highlights the need for lifetime follow-up in patients treated for NPC.

Our clinical series is focused on relapsed NPCs treated with curative intent in a non-endemic area. As in the curative setting (20, 21), we observed a clear dominance of the non-keratinizing undifferentiated carcinoma (86.2%). Given the more favorable biological profile, non-keratinizing tumors are probably more prone to give treatable relapses, unlike the keratinizing subtypes which behave more aggressively (20).

T categories at recurrence were distributed homogeneously, with similar rates of rcT from 1 to 4. Furthermore, **Table 2** shows that a substantial proportion of recurrent cases (53 of 123, or 46.3%) maintained the T category displayed at presentation. However, several cases were classified with a rT category different to that at presentation. Of note, 22.1% of patients had a nodal recurrence, with only 7 (5%) patients showing isolated nodal recurrence.

The choice of the therapeutic strategy for each patient was based on multiple factors, including the interval after previous RT, morbidity of primary treatment, staging, burden of disease, and experience of the multidisciplinary team, along with general conditions and motivation of the patient.

In the present cohort, all the contemporary curative therapeutic options are represented. The documents guiding the clinical practice, that were cited in the present paper, were mostly focused on endemic populations, since specific prospective trials and guidelines on non-endemic cohorts are lacking. However, when we compare our curative patterns of care with those from endemic regions, we did not find relevant differences. Indeed, in a work by Ng et al. (19) including 272 locally recurrent NPC patients treated in six Hong Kong public hospitals, radical surgery with or without adjuvant RT or chemotherapy (classified as the surgery group) and re-RT with or without induction or concurrent chemotherapy (classified as the re-RT group) were administered with a similar percentage.

Of 140 patients, about 20% underwent neoadjuvant chemotherapy, with a clinical benefit rate (i.e., at least a stable disease) of 80%. The role of induction chemotherapy for recurrent NPC is still debated (22), with the literature lacking in strong evidence. Even in the absence of clear guidelines, the multidisciplinary team may propose selected patients for neoadjuvant chemotherapy in light of the beneficial effects on progression-free survival, OS, loco-regional and distant control observed in patients with primary NPC (23). Furthermore, in cases that are not suitable for re-RT or surgery, it may shrink the tumor and regain the potential for further surgical or radiation treatments, with better outcomes than those observed after chemotherapy (24).

About half of patients underwent surgery on T and/or N. In line with the literature from endemic areas (19), the most common treatment modality for low-stage tumors was surgery, while T4 lesions represented 42.6% of all the lesions treated with re-RT (vs. 5% of the surgical group). Age and CCI were lower in the surgical group, suggesting that selection of patients with favorable conditions did occur. Furthermore, patients with short DFI preferentially underwent surgical resection (**Table 4**). This is consistent with the evidence that short DFI is associated with lower control rate and higher morbidity if re-RT is indicated (6, 25).

NER represented the surgical approach of choice in all locally recurrent NPC, achieving free resection margins in 85.2% of patients. The preference towards endoscopic approaches was influenced by the many experiences from the literature reporting that survival and morbidity outcomes with a transnasal route are significantly superior to those of open surgery (26). Of note, surgeons operating in non-endemic areas have contributed significantly in the developement and refinement of endoscopic approaches, showing good results on this population (16).

The remaining half of the patients were treated with re-RT, distributed between photon-based RT, with either IMRT or SRT, and PT (54.4% vs. 42.8%, respectively).

All patients in our re-RT series received at least 45Gy/GyRBE, which is historically considered a total dose that is able to control recurrent disease, although recently international recommendations suggest giving at least 60 Gy (6). So far, although patients with small and potentially resectable recurrences could be efficiently treated with SRT, there is general consensus in using photon-based IMRT or PT, in particular for larger recurrent diseases (6). Due to the paucity of PT facilities worldwide, the choice between IMRT and PT should be ideally based on dosimetric comparison, so that resources are rationally utilized. As in treatment-naive patients, recent publications demonstrated that PT has some dosimetric advantages over IMRT in treating recurrent NPC (27, 28).

In the literature, a wide range of different survival rates is reported for recurrent NPC (9, 19, 29), reflecting the heterogenous stage distribution and treatment modalities. With 29 months median duration of follow-up, we observed a 5-year OS, DSS, and RFS estimates of 55.9, 62.1, and 41.3%, respectively. Our results set at an intermediate level in the wide range of outcomes, with surgical clinical series focusing on low-stage tumors reporting better results [73.8% 5-year OS in Liu et al. (9)] and studies on high-stage recurrent NPC treated with re-RT showing fewer encouraging results [37.0% 5-year OS in Boustani et al. (14)]. This owes to the fact that the full spectrum of stages was included in the present series.

Older patients experienced worse OS and DSS, in line with the literature (1). A shorter DFI influenced positively OS and DSS. Conversely, Tian et al. reported a negative effect on survival in patients whose disease recurred within 24 months from primary treatment (30). In the meta-analysis by Yue et al., however, no significant association was observed between recurrence time interval and OS (31). Our results could be justified by the non-negligible proportion of patients treated with surgery. A short DFI can be considered as an indirect sign of radio-resistance, thus directing the choice of treatment towards surgery. In case of persistence or short-DFI recurrence, an R0 nasopharyngectomy is held to eliminate radioresistant clones, improving survival. On the other hand, re-RT of an early recurring, radiosensitive NPC may correct an inadequate RT dose and/or dose distribution during primary treatment. In both scenarios, early salvage treatment can be considered as completion to primary treatment. Conversely, late relapsing NPCs have several potentially adverse features, including development of secondary cancer, asymptomatic growth to advanced stage, and reduction of patient’s global reservoir to receive aggressive treatment.

Many research groups have confirmed the impact of rT classification on survival outcomes, both in surgical and re-RT series (1, 29). Accordingly, at our univariable analysis, primary and recurrent T category significantly influenced OS, DSS, and RFS. However, despite the increasing HR from rcT1 to rcT4, this association was not confirmed at multivariable analysis. RFS after recurrent tumor treatment was worse in cases with nodal involvement. Interestingly, these patients showed further recurrences more frequently on the primary sites instead of neck lymph nodes. This could imply rcT0N+ NPCs are associated with a considerable probability of occult local recurrence that becomes clinically appreciable after a certain time.

Although the RT group was predominantly represented by patients with rT3-4 cancers, no independent prognostic effect of loco-regional treatment modality (definitive re-RT vs. surgery w/o adjuvant therapy) on OS, DSS, or RFS was observed at multivariable analysis. This is partially inconsistent with the literature (9, 19). In our real-world series, the selection of the treatment modality was affected by technological availability together with specific experience of local radiation oncologists. Furthermore, the non-endemic nature of NPCs included in this study might have influenced the biological behavior and response to therapies.

Surgical margin status did not affect OS at univariable analysis. Furthermore, at multivariable analysis, no difference was highlighted between R0 and R1 resections in terms of OS, DSS, and RFS. Being prone to many biases, margin assessment in NER is probably a not reliable estimate of microscopic residual disease. The use of electrocautery or laser leads to shrinkage of the surgical specimen and hampers final pathological analysis. Moreover, the cancer advancement front may be non-homogenous: the effects of primary RT and neoadjuvant chemotherapy are different on cancer cell populations, resulting in potential satellite clones far from the main tumor advancement front (10). Recent consensus guidelines and literature data have reported that opinions vary widely from liberal use of postoperative re-RT to PT in case of involved surgical margins (6, 26). In the present series, according to local multidisciplinary team discussion, re-RT was indicated in case of margin involvement or advanced stage, but the main constraining factor was represented by cumulative dose distribution and estimated risk of toxicity. Thus, positive margins were not always associated with the chance of re-RT, nor did negative margins imply adjuvant re-RT to be automatically excluded from salvage treatment.

Neoadjuvant and/or adjuvant chemotherapy showed a protective effect on RFS, though not determining an impact on OS and DSS. The decreased probability of recurrence is probably counterbalanced by the chemoselection of aggressive clones, so that further recurrences, even if rarer than in patients not receiving chemotherapy in the salvage treatment, might display a more aggressive behavior. On the other hand, one cannot rule out selection bias of treating patients with a higher burden of disease by chemotherapy, and the possibility that in patients who did not receive chemotherapy, its subsequent administration at further relapse may have counterbalanced the positive effect on OS. However, the multistate analysis revealed that chemotherapy had no impact on transitions to death. Overall, chemotherapy showed a positive effect in terms of disease control, but its role in recurrent NPC still needs to be fully elucidated.

We did not find a difference in terms of survival in patients treated with different techniques of re-RT (IMRT/VMAT, SRT, and PT). This might be partly due to the limited cohort size.

In our series, 40% of patients experienced at least one adverse event classified as G3 or higher. As seen at multivariable analysis, age and DFI were independent factors influencing OS. It is likely that toxicity of treatment heavily contributed to this observation. Indeed, multivariable multistate analysis showed that the same covariates were associated with an increased risk of transitioning from a state of ≥G3 toxicity to recurrence-free death. Of note, ≥G3 toxicity occurred constantly over the post-retreatment period, thus underlying the need for close surveillance of these possible adverse effects (**Figure 4**).

The most frequent late complication in both the surgical and re-RT groups was skull base osteomyelitis. Schreiber et al. demonstrated the correlation between the location and entity of the osteomyelitis with the field and dose of RT (32). This justifies the finding that its frequency is remarkably higher in the re-RT group compared to the surgical group (10 vs. 6 cases, respectively) (22). However, this observation might be related to the fact that the re-RT group included a higher proportion of high-stage diseases. Patients with most advanced recurrences are hardly ever candidates for surgery and usually undergo re-RT, with higher risk of morbidity. Moreover, since high-stage primary lesions are prone to have high-stage recurrence (**Table 2**), re-irradiated patients had likely already undergone a primary irradiation with a non-negligible toxicity profile.

Of note, all 3 cases of internal carotid artery blowout observed in the re-RT group were treated with PT. In comparison to conformal RT techniques at the same prescription dose, PT planning can result in very high dose hot spots in the target volume, with a potentially high rate of vascular and mucosal complications (27). Moreover, although re-RT planning criteria to ensure both tumor coverage and organs at risk preservation have been proposed with the goal of decreasing radiation-induced life-threatening injuries (thus increasing cure rates) (6, 33), a comprehensive dosimetric analysis of normal tissue complications with more advanced RT techniques, including PT, is lacking. In selected cases, the stenting or occlusion of the carotid artery before starting salvage treatment should be considered to avoid fatal blowouts, although the chance of cerebrovascular and non-cerebrovascular complications should be discussed within the multidisciplinary team (11).

No significant difference in the morbidity profile of treatments was observed. In fact, not only was the rate of ≥G3 adverse events not significantly different among treatment strategies, but multistate multivariable analysis also showed that there was no impact of treatment modality on the transition from the disease- and toxicity-free state to recurrence-free ≥G3 toxicity state. This discrepancy with the literature, where surgery is associated with lower complication rates (9), can be partially explained by the adverse events classification system used in this study. Even if designed to study the adverse effects of RT, the CTCAE system allows for homogeneous and fair assessment of complications and unveiled a more balanced situation between the treatment arms as would have been expected. Of note, in the surgical group, reconstruction with a vascularized flap showed a protective effect on the development of ≥G3 toxicity, thus highlighting the importance of using vascularized tissue to ensure adequate blood supply to previously irradiated tissues, which by definition have impaired microcirculation.

Finally, we provided a prognostic nomogram for non-endemic recurrent NPC, which had adequate accuracy at internal validation. Sun et al. (34) developed a nomogram for patients with endemic local recurrent NPC based on pre-treatment data, thus allowing prediction of OS and guide individualized treatment. We chose to include data in the pre- and post-treatment phases, so that the nomogram can be used: 1) to have a quantitative estimate of chances of survival in a given patient and 2) to appreciate the putative effect of controllable variables (e.g., treatment strategy, use of vascularized reconstruction) on the overall outcome estimate.

The main limits of this study are represented by its retrospective nature and the collection of multiple experience of different referral centers, sharing the same philosophy but not following a common treatment protocol. The precise collection of other recognized prognostic factors, such as performance status, quality of life, and circulating Epstein-Barr virus DNA, was not possible in this retrospective series. Furthermore, the analyses included local, regional, and locoregional recurrences. This makes the results of the present study more complex to be interpreted but brings them closer to a real-world scenario. Overall, taking into consideration the heterogeneity and the rarity of the pathology, this study can be considered as the basis for future prospective trials.

## Conclusion

To date, clear evidence guiding the choice of treatment for non-endemic recurrent NPC are lacking. In our series, favorable cases with lower age, comorbidity rate, and stage underwent preferentially endoscopic surgery, as well as patients with shorter DFI from primary treatment. More complex cases underwent re-RT, distributed between photon-based RT and PT.

Age and DFI were independent factors influencing OS. No independent prognostic effect of treatment modality was observed, suggesting that the non-endemic nature of NPCs might have influenced the biological behavior and response to therapies. No statistical difference in the morbidity profile of treatments was observed, with 40% of patients experiencing at least one adverse event classified as G3 or higher.

The recurrent non-endemic NPC has dissimilar aspects compared to the endemic counterpart, suggesting the need for further survival studies that can guide the choice of the best treatment modality for each patient.

## Data availability statement

The raw data supporting the conclusions of this article will be made available by the authors, without undue reservation.

## Ethics statement

The studies involving human participants were reviewed and approved by Comitato etico di Brescia, Brescia, Italy. The patients/participants provided their written informed consent to participate in this study.

## Author contributions

VR and MF substantially contributed to the conception and design of the work, interpretation of data, and drafting the work. DM, PiB, AL, MT-Z, ED’A, DA, MC, BV, MT, RR, MA, ST, DT, MM, MB, NI, FD, MaBi, AD, PaBa, PaB, and CP substantially contributed to acquisition and interpretation of data. AS, PN, PC, and EO substantially contributed to conception and design of the work, and interpretation of data. All Authors provide approval for publication of the content and agree to be accountable for all aspects of the work in ensuring that questions related to the accuracy or integrity of any part of the work are appropriately investigated and resolved.
